# A rectosigmoid haemangioma: A rare etiology of rectal bleeding

**DOI:** 10.1259/bjrcr.20220084

**Published:** 2022-09-12

**Authors:** Shareefa Abdulghaffar, Muna AlMulla, Dana AlNuaimi, Reem AlKetbi, Tarig ElNour Khairi

**Affiliations:** 1 Department of Radiology, Dubai Health Authority, Dubai, United Arab Emirates

## Abstract

Cavernous haemangiomas of the rectosigmoid colon are rare benign vascular neoplasms of the GI tract. Patients usually present at a younger age with various degree of rectal bleeding ranging from mild painless episodic bleeding to life-threatening hemorrhage. High index of suspicion and early diagnosis is crucial to avoid unnecessary biopsy and inappropriate management. We report a case of a 26-year-old male patient with a long history of recurrent rectal bleeding. Contrast-enhanced CT scan of the abdomen and pelvis and MRI confirmed the diagnosis of cavernous hemangioma. Further surgical treatment with rectosigmoid resection and colo-anal anastomosis represents the optimum path of management for our patient.

## Introduction

Intestinal haemangiomas are rare benign vascular malformations that account for about 0.05% of all gastrointestinal tumors with an incidence rate of 1 in every 1500 patients.^1,2^ It is usually seen in young adults and presents with painless episodic rectal bleeding.^3^ The rectosigmoid colon is the commonest site of involvement by cavernous hemangiomas in the gastrointestinal tract.^4–6^ The diagnosis is usually often delayed due to physician’s low index of suspicion and the majority of cases are misdiagnosed as internal hemorrhoids or inflammatory bowel disease.^3^ Therefore; clinicians and radiologist should be aware of this entity to avoid misdiagnosis and delayed or inappropriate management.

## Case report

A 26-year-old Middle Eastern male patient presented to the outpatient gastroenterology clinic complaining of recurrent episodes of fresh bleeding per rectum for the past 11 years. Bleeding is not associated with defecation and the amount of blood is variable. At times, it is associated with abdominal pain and loose stools. The patient gave a surgical history of undergoing surgical treatment of a rectal vascular malformation at the age of 8 years. No other significant medical history was given.

On physical examination, the patient vitals were within the normal limits. Chest was clear and abdomen was soft to palpation with evidence of a transverse scar as well as a colostomy scar suggestive of previous surgery. Per rectal examination denoted multiple nodularities in the rectum and fresh blood was observed. Laboratory blood work-up revealed a picture of microcystic anaemia and occult blood stool was positive.

**Figure 1.**
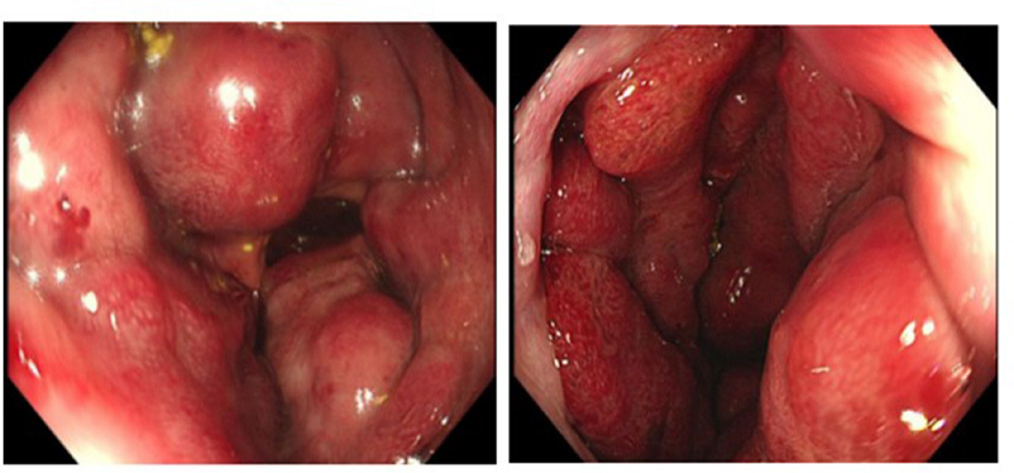
Multiple congested huge swellings with erythematous mucosa in the rectum.

Colonoscopy showed multiple congested large swellings extending 7 to 15 cm from the anal verge with normal appearing mucosa above that level suspicious for a vascular aetiology thus no biopsy was taken (Figure 1). Upper GI endoscopy showed mild gastritis with no suspicious lesions notable.

**Figure 2.**
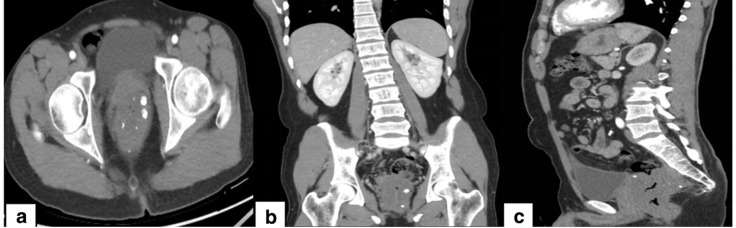
Contrast-enhanced CT of the abdomen and pelvis in 5-mm axial sections (**A**) with coronal and sagittal reformatted images (B & C, respectively) showing diffuse circumferential thickening of the rectal wall with perirectal serpiginous structures and chunky calcifications likely representing phleboliths. No evident contrast enhancement was seen.

Contrast-enhanced computed tomography of the abdomen and pelvis showed nodular chunky calcifications with central lucencies likely representing phleboliths with diffuse circumferential thickening of the recto-anal wall and a right-sided perirectal serpiginous structure likely of vascular origin. Nonetheless no contrast enhancement was appreciated. The rest of the bowel appeared grossly normal. No evidence of bowel obstruction was seen. Solid organs appeared normal. Focal nodal calcification was seen in the small bowel mesentery likely representing an old healed inflammatory or infectious process. (Figure 2)

**Figure 3.**
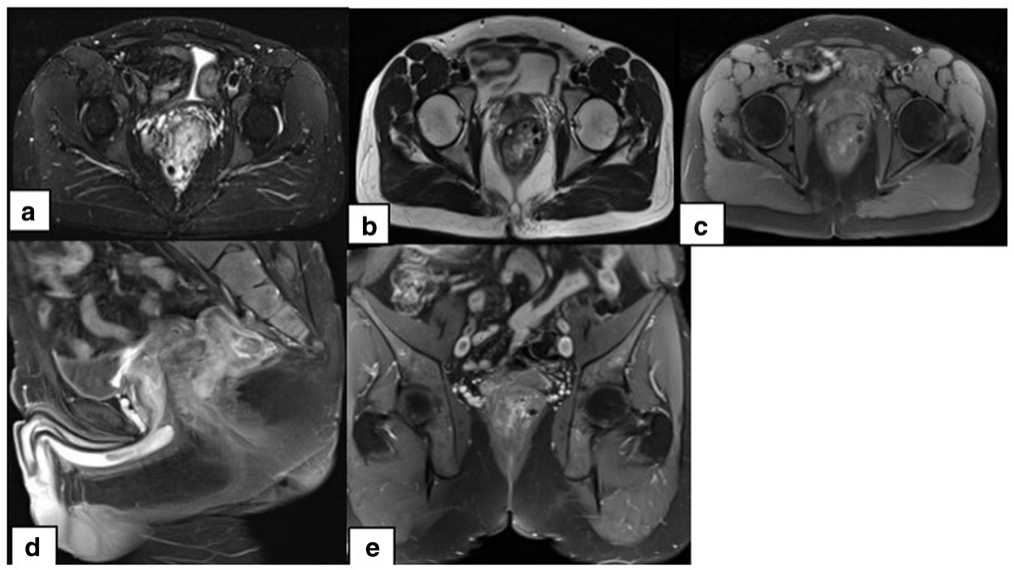
Gadolinium-enhanced MRI of the pelvis in A. Axial T1 with contrast, B. Axial T2, C. Axial STIR, D. Sagittal FATSAT with contrast and E. Coronal T1 with contrast revealing circumferential submucosal thickening of the rectum with heterogenous enhancement and multiple signal voids likely representing calcifications.

Gadolinium-enhanced magnetic resonance imaging of the abdomen and pelvis revealed circumferential submucosal thickening of approximately 7 cm in length up to 2.5 cm from the anal verge appearing low on T1 sequences and high on T2 sequences with heterogenous enhancement and multiple signal voids suggestive of calcifications. No evidence of restricted diffusion. Multiple prominent enhancing vessels in the post-contrast images were noted. The prostrate and seminal vesicles were slightly displaced due to the rectal wall thickening. Urinary bladder appears normal with no evidence of free fluid or pelvic collection. (Figure 3)

**Figure 4.**
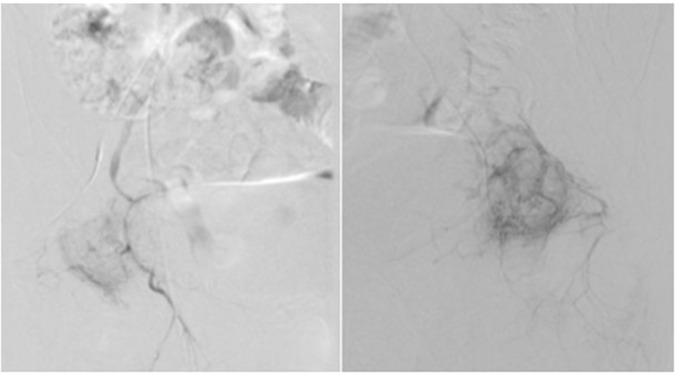
Selective abdominal angiography showing the middle rectal artery feeding the rectal haemangiomas (blue arrow).

A digital subtraction angiogram of the internal iliac arteries identified prominent middle rectal artery branches supplying the rectal wall swellings (Figure 4). Nonetheless, sclerotherapy was not attempted during the procedure. The patient was referred for surgical resection and colo-anal anastomosis.

## Discussion

Haemangiomas are benign vascular lesions that can involve any part of the body including the gastrointestinal tract.^1,2^ Intestinal haemangiomas are rare accounting for only 0.05% of all the GI tumors and usually present in children and young adults with history of painless, recurrent rectal bleeding and anaemia.^2,4^ The rectosigmoid colon is the commonest site of involvement accounting for 50–70% of the cases.^4,5^Intestinal haemangiomas are usually solitary and occasionally associated with certain syndromes such as Osler-Weber-Rendu syndrome, Maffucci syndrome and Klippel Trenaunay-Weber syndrome.^6^ There are three types of haemangiomas: cavernous, capillary and mixed haemangiomas with cavernous hemangiomas being the commonest type.^1^

Due to rarity of intestinal haemangiomas these lesions are usually misdiagnosed in 80% of cases for other commoner etiologies of lower GIT bleeding including internal hemorrhoids, inflammatory bowel disease and colorectal cancer.^1,2^ The gold standard diagnostic tool of choice is video-colonoscopy as it is a non-invasive technique that requires no radiation and which accurately localises and depicts the morphology and size of the lesion as well as excludes other synchronous lesions.^1^ On colonoscopy, cavernous haemangiomas appear as solitary or cluster of bluish or red submucosal soft lesions that are easily compressible on insufflation with adjacent engorged veins in the rectal wall.^2,3^ Due to the vascular nature of the lesion, biopsy is not recommended.^5^

Abdominal radiographs usually show multiple scattered pelvic phleboliths which represent thrombosed and calcified intralesional vascular channels.^3^ CT scan of the pelvis can confirm the diagnosis as it shows concentric thickening of the rectosigmoid colon with multiple intralesional phleboliths and heterogeneously enhancing perirectal soft tissues.^4,6^ Due to risk of radiation from multiple follow-up scans on these young patients, MRI is a suitable alternative imaging modality. Rectosigmoid haemangiomas appear as wall thickening with high T2 signal intensity due to slow flow voids within the vascular malformations and progressive nodular enhancement on post-contrast images.^6^ Moreover, *T_2_*-weighted images aid in differentiating colorectal carcinoma from haemangiomas which usually show intermediate signal on T2 images in comparison with the adjacent high signal intensity rectal fat and low signal intensity colonic muscular layer.^4^

Surgical resection is reserved for larger and diffuse rectosigmoid haemangiomas, while smaller submucosal haemangiomas can be resected endoscopically.^7^ Abdominoperineal resection (APR) was considered the standard treatment previously, however due to the resultant permanent stoma and common complications such as sexual and urinary dysfunction, it is currently a less desirable approach.^8^ Advocated colo-anal sleeve anastomosis is instead the new popular approach that allows resection of the lesions without a permanent stoma.^8^ DCHR may not be completely resolved by non-operative therapies such as sclerosing injections, microwave treatment, and trans-fixation or ligation of the internal iliac artery and recurrent rectal bleeding is often common after these treatments.^8^

## Conclusion

We reported a case of a 26-year-old male patient with rectosigmoid hemangioma. While this entity is a rare cause of rectal bleeding, it should be kept in the differential diagnosis especially in young patients presenting with a long history of episodic hematochezia. Video colonoscopy is the gold standard diagnostic tool and CT and MR imaging play a crucial role in confirming the diagnosis and evaluating the extent of the disease prior to surgical management.

## Learning points

- Proper evaluation of diffuse cavernous haemangiomas with diagnostic imaging modalities is necessary while investigating patients with rectal bleeding in order to reach a definitive diagnosis.
- Colonoscopy and MRI are cornerstones for the correct diagnosis of rectal cavernous haemangiomas.
- Bleeding is the most feared complication because of the high vascularity of the rectal cavernous haemangiomas.
- Careful assessment of the whole of the gastrointestinal tract is needed to rule out diffuse involvement.
- Surgical resection is the definitive management.
